# Retinal Venular Tortuosity Jointly with Retinal Amyloid Burden Correlates with Verbal Memory Loss: A Pilot Study

**DOI:** 10.3390/cells10112926

**Published:** 2021-10-28

**Authors:** Oana M. Dumitrascu, Ryan Rosenberry, Dale S. Sherman, Maziyar M. Khansari, Julia Sheyn, Tania Torbati, Ayesha Sherzai, Dean Sherzai, Kenneth O. Johnson, Alan D. Czeszynski, Steven Verdooner, Keith L. Black, Sally Frautschy, Patrick D. Lyden, Yonggang Shi, Susan Cheng, Yosef Koronyo, Maya Koronyo-Hamaoui

**Affiliations:** 1 Department of Neurology, Mayo Clinic, Scottsdale, AZ 85251, USA; 2 Department of Cardiology, Cedars-Sinai Medical Center, Los Angeles, CA 90048, USA; ryan.rosenberry@cshs.org (R.R.); susan.cheng@cshs.org (S.C.); 3 Department of Neuropsychology, Cedars-Sinai Medical Center, Los Angeles, CA 90048, USA; dale.sherman@cshs.org; 4 USC Stevens Neuroimaging and Informatics Institute, Keck School of Medicine of University of Southern California, Los Angeles, CA 90007, USA; Maziyar.Khansari@loni.usc.edu (M.M.K.); Yonggang.Shi@loni.usc.edu (Y.S.); 5 Department of Neurosurgery, Cedars-Sinai Medical Center, Los Angeles, CA 90048, USA; julia.sheyn@cshs.org (J.S.); tania.torbati@westernu.edu (T.T.); keith.black@cshs.org (K.L.B.); yosef.koronyo@cshs.org (Y.K.); 6 Department of Neurology, Loma Linda University, Loma Linda, CA 92350, USA; Asherzai@llu.edu (A.S.); dsherzai@llu.edu (D.S.); 7 NeuroVision Imaging Inc., Sacramento, CA 95833, USA; kjohnson@neurovision.com (K.O.J.); aczeszynski@neurovision.com (A.D.C.); sverdooner@neurovision.com (S.V.); 8 Department of Neurology, University of California Los Angeles, Los Angeles, CA 90095, USA; sfrautschy@mednet.ucla.edu; 9 Department of Neurology, Cedars-Sinai Medical Center, Los Angeles, CA 90048, USA; plyden@usc.edu; 10 Department of Biomedical Sciences, Cedars-Sinai Medical Center, Los Angeles, CA 90048, USA

**Keywords:** retinopathy, retinal vessels, retinal fluorescence imaging, amyloid, cognitive decline, Alzheimer’s disease

## Abstract

Introduction: Retinal imaging is a non-invasive tool to study both retinal vasculature and neurodegeneration. In this exploratory retinal curcumin-fluorescence imaging (RFI) study, we sought to determine whether retinal vascular features combined with retinal amyloid burden correlate with the neurocognitive status. Methods: We used quantitative RFI in a cohort of patients with cognitive impairment to automatically compute retinal amyloid burden. Retinal blood vessels were segmented, and the vessel tortuosity index (VTI), inflection index, and branching angle were quantified. We assessed the correlations between retinal vascular and amyloid parameters, and cognitive domain Z-scores using linear regression models. Results: Thirty-four subjects were enrolled and twenty-nine (55% female, mean age 64 ± 6 years) were included in the combined retinal amyloid and vascular analysis. Eleven subjects had normal cognition and 18 had impaired cognition. Retinal VTI was discriminated among cognitive scores. The combined proximal mid-periphery amyloid count and venous VTI index exhibited significant differences between cognitively impaired and cognitively normal subjects (0.49 ± 1.1 vs. 0.91 ± 1.4, *p* = 0.006), and correlated with both the Wechsler Memory Scale-IV and SF-36 mental component score Z-scores (*p* < 0.05). Conclusion: This pilot study showed that retinal venular VTI combined with the proximal mid-periphery amyloid count could predict verbal memory loss. Future research is needed to finesse the clinical application of this retinal imaging-based technology.

## 1. Introduction

By 2025, the number of people aged 65 years and older with Alzheimer’s dementia (AD) is projected to reach 7.1 million, which is almost a 22% increase from 2020 [1,2]. The contribution of vascular disease to cognitive performance is increasingly recognized, as the mechanisms linking vascular dysfunction and neurodegeneration are better characterized [3,4,5,6,7]. Recent reports implicate cerebral vascular pathology as an early and core contributor to the development of not only vascular dementia but also AD [7,8,9,10,11], a neurodegenerative condition and looming public health threat [2,12]. Considering the emerging vascular hypothesis [8,13,14,15], there is a critical need to incorporate both vascular and AD biomarkers [16,17,18,19,20] into predictive models to allow for early and sensitive detection of AD and mixed dementias. Yet, imaging of the skull-shielded brain poses various limitations for widespread screening in the clinical setting. The retina is a central nervous system organ that exhibits Aβ deposition and vascular changes [21,22,23,24,25,26,27,28,29,30,31,32,33] and is far more accessible for repeated and high-resolution imaging [34,35,36,37,38,39,40,41,42]. Dysfunctional pericytes in the blood-brain barrier (BBB) are significant contributors to the pathogenesis of vascular cognitive impairment, including cerebral small vessel and cerebral large vessel disease, as well as AD [28,43]. BBB pericyte injury is a predictor of apolipoprotein E (APOE) ε4-associated cognitive decline [4]. In contrast, BBB dysfunction mediates cerebral Aβ deposition, the retinal–blood barrier mirrors the BBB, and its disruption in the form of retinopathy was shown to predict cognitive decline [28,44,45,46,47,48,49]. Post-mortem retinal vessels derived from patients with mild cognitive impairment (MCI) and AD exhibited early and progressive pericyte loss as well as Aβ accumulation inside retinal pericytes, correlating with similar AD pathology in the brain [28]. Several studies demonstrated the linkage between retinal vascular fractal dimensions, caliber, and both tortuosity and cognitive deterioration [45,46,47,48,49,50,51]. The retinal arteriolar central reflex to vessel width ratio in digital retinal photographs was significantly higher in APOE ε4 allele carriers [48], hence the retina may allow for non-invasive monitoring of the effects of APOE ε4 on the cerebrovascular disease. Similarly, as targeting vascular risk factors is being considered in dementia prevention trials [52], retinal vascular assessments could offer a window for assessing the response to various interventions.

Recent work has highlighted the promising utility of retinal fluorescence imaging, an emerging technique capable of non-invasively imaging and quantifying the retinal amyloid, which is the pathological marker of AD [22,23,26,34,53,54,55]. Using this technique, our group previously identified a significant association between retinal amyloid count, especially in the proximal mid-periphery area, and the severity of cognitive impairment as well as hippocampal volumes [34,35]. As the same retinal imaging modality also allows for retinal vasculature analysis, we aimed to quantitatively examine both retinal vascular and retinal amyloid biomarkers in a cohort of subjects with cognitive decline. In this proof-of-concept exploratory study, we sought to examine the relationship between retinal microvascular features and retinal amyloid burden, with global and domain-specific cognitive scores.

## 2. Materials and Methods

### 2.1. Participants

This pilot study was approved by the Cedars-Sinai Institutional Review Board. All subjects older than 40 years of age presenting to our Neurology clinic with subjective cognitive decline and interest in undergoing retinal fluorescence imaging were included in this cohort. All subjects underwent a neurological examination, a standard battery of neuropsychological tests, and standard-of-care 3 Tesla non-contrast structural brain magnetic resonance imaging (MRI). No exclusion criteria were prespecified, except for a history of glaucoma, allergy to mydriatic eye drops, curcumin, or vitamin E. All subjects provided written informed consent prior to the commencement of the study.

### 2.2. Retinal Imaging

**Figure 1:**
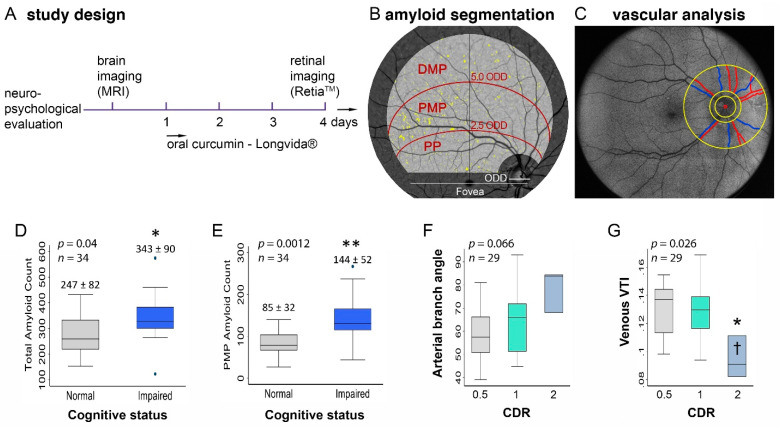
Study timeline of brain and retinal imaging followed by sectoral amyloid and vascular analysis. Study design scheme illustrating that subjects underwent baseline brain imaging and neuropsychological evaluation, followed by retinal fluorescence imaging after 4 days of daily oral curcumin intake (**A**). Illustration of the region of interest in the right eye supero-temporal retinal quadrant and its three subregions, which were used for quantifying retinal amyloid counts (**B**). Illustration of the region of interest used for the retinal vascular analysis. The red circle indicates the center of the optic nerve-head and the smallest yellow circle shows the optic nerve-head area. The two larger circles indicate the region of interest for the vascular analysis, which were 1.5 and 4 times the diameter of the optic disc. The branching angle and tortuosity of vessels within the region of interest were calculated. Arteries and veins are outlined by red and blue lines, respectively (**C**). Graphs illustrating differences in total amyloid (**D**) and proximal amyloid counts (**E**) when stratified by cognitive status. Graphs illustrating the differences between arterial branching angle (**F**) and the venous tortuosity index (**G**) when stratified by CDR. * *p* < 0.05; ** *p* < 0.01, by two-tailed unpaired student *t*-test or one-way ANOVA and Bonferroni’s post-hoc test. Abbreviations: MRI, magnetic resonance imaging; PP, posterior pole; PMP, proximal mid-periphery; DMP, distal mid-periphery; ODD, optic disc diameter; CDR, Clinical Dementia Rating; and VTI, vessel tortuosity index.

After ocular dilation, the retinal imaging was performed with a confocal scanning ophthalmoscope (Retia^TM^, CenterVue SpA) that utilizes blue light for the excitation of curcumin emission to obtain fluorescent images of the retina, following a study design described in prior reports (Figure 1A) [34,35]. Curcumin has high affinity and specificity for the β-pleated sheets of Aβ, specifically for Aβ42, oligomers, and fibrils, which are linked to AD [56,57,58,59,60,61]. The researchers conducting the retinal image processing and quantifications were blinded to the patients’ clinical characteristics.

### 2.3. Retinal Amyloid Quantification

The set of retinal images were processed using an automated retinal fluorescence measurement software system (NeuroVision Imaging, Inc., Sacramento, CA, USA). A combination of algorithms, including background correction, followed by characterization of the corrected retina using a mixture model, were used to identify pixels that were abnormally bright. Specifically, the primary factor of the variation in pixel intensity is illumination variability across the entire field of view (e.g., edges of the image become dark). This variability is addressed by estimating the background level and correcting it. The secondary factor in pixel variability is the structure to which it depends on. Vessels appear dark or hypofluorescent, while the amyloid appears identically as bright or hyperfluorescent. The background correction produces all vessels at a more consistent pixel value. In a similar manner, this occurs for the retina and amyloid spots. Choosing the appropriate threshold is possible using the mixture model, which characterizes hypofluorescent, isofluorescent, and hyperfluorescent pixels appropriately. A common region of interest (ROI) in the supero-temporal quadrant was applied with a field of view of 50 degrees, positioned on the image center using fovea and optic nerve-head centers as reference points to correct for eye rotation, with a zone around the fovea and optic nerve-head masked, as previously reported [35]. The ROI was further divided into three subregions: posterior pole, proximal mid-periphery, and distal mid-periphery (Figure 1B). Retinal amyloid count was quantified in the target ROI and three specified subregions.

### 2.4. Retinal Vascular Quantification

From the same retinal fundus images, an ROI was defined within a circumpapillary region centered on the optic nerve-head (ONH) and extending between 1.5 and 4 ONH radii (Figure 1C) [62]. Before the analysis, retinal images were visually inspected to ensure vessels were visible and that there was no reflectivity that could influence the result. The major vessels were detected after intensity normalization to minimize the effect of other influencing factors. Retinal vessels within the ROI were segmented using the Frangi vesselness filter to generate a binary image [63]. The vessels were classified into arteries and veins by a human observer based on the facts that retinal arteries are brighter in color and thinner in width compared to veins [64]. For each vessel segment on the binary image, vessel endpoints were selected, and distance transformation was used to extract the vessel centerline. The extracted centerlines were smoothed using a cubic spline with a regularization parameter of 3 × 10^−5^. For each centerline, several geometric features, including the vessel tortuosity index (VTI), vessel inflection index, and branching angle, were non-automatically quantified. The VTI was calculated for each centerline based on a combination of local and global centerline geometric variables, as explained previously, that can detect alterations in the retinal vessels’ curvature with pixel-level accuracy [65]. Equation (1) shows the formula for the VTI.

(1) VTI=0.1×(SDθ.N.M.LALC)

where ${\mathrm{SD}}_{\theta }$ the is standard deviation of the angle difference between lines tangent to each centerline pixel and a reference axis (i.e., x-axis), and M is the average ratio of the centerline length to its chord length between pairs of inflection points, including centerline endpoints. N is number of critical points where the first derivative of the centerline vanishes, while L_A_ and Lc are the length of the vessel and its chord length, respectively. The VTI is shown to provide good correspondence with human perception of tortuosity and is invariant to rigid transformations. Similar to other measures of tortuosity, VTI is unitless. Its minimum value is zero, while it has no theoretical maximum as it can increase with the twistedness of a vessel. The vessel inflexion index was determined based on a number of inflection points along the vessels. Mathematically, these were pixels where the second derivative of the centerline vanishes. The vessel inflexion index represents local changes in the tortuosity of vessels and was found to be robust for ranking the tortuosity of vessels with similar lengths [66]. The branching angle of the vessels was calculated interactively using the open-source tool GIMP 2.8.

### 2.5. Cognitive Evaluation

All participants underwent a standard battery of neuropsychometric testing performed by a licensed neuropsychologist (DS). Standard neuropsychological testing included the Montreal Cognitive Assessment (MOCA), global Clinical Dementia Rating (CDR), as well as general cognitive (ACS-test of Premorbid Functioning) and specific cognitive domain assessments: attention and concentration (Wechsler Adult Intelligence Scale (WAIS)-IV); verbal memory (California Verbal Learning Test (CVLT) II, Wechsler Memory Scale (WMS)-IV, and Logical Memory II); non-verbal memory (Rey Complex Figure Test and Recall (RCFT) 30 min, and Brief Visuo-Spatial Memory Test Revised (BVMT-R) Delayed Recall); language (Fluency-Letter (FAS) and Fluency-category (animals)); visuo-spatial ability (Rey Complex Figure Test and Recognition Trial (RCFT) Copy); speed of information processing (Trails A and B); and symptom validity and functional status (SF-36 Physical Component Score (PCS) and Mental Component Score (MCS)). We also evaluated the subject’s emotional status using the Beck Depression Inventory II, Geriatric Depression Scale, and Profile of Mood State/Total Mood Disturbance.

### 2.6. Statistical Analysis

Descriptive statistics were calculated for patient demographics and clinical characteristics. Unless otherwise stated, data are expressed as mean ± standard deviation. Subjects were partitioned into three groups according to the Clinical Dementia Rating (CDR) (0.5, questionable impairment; 1, mild cognitive impairment; and 2, moderate cognitive impairment) [67] and dichotomized using MOCA, which demonstrates excellent sensitivity and specificity for both mild cognitive impairment (MCI) and AD. Using the cutoff score of <26, the MOCA has excellent sensitivity for MCI (90%) and AD (100%), as well as for the specificity for normal controls (87%). Positive (PPA) and negative predictive accuracy (NPA) were also reported to be excellent with a PPA of 89% and NPA of 91% for MCI, and a PPA of 89% and NPA of 100% for AD [68]. The subjects were also partitioned into groups according to the neuropsychometric diagnosis (normal cognition versus impaired cognition).

To produce combined indices of retinal vascular and amyloid measures, each variable was first inspected for normality; any non-normal variables were then log-transformed to produce a normal distribution. Each normalized variable was then standardized to a mean of 0 and a standard deviation set equal to 1. While higher amyloid count was associated with worse cognitive function, higher venous vascular tortuosity index (VTI) values were associated with better cognitive function. To account for this inverted scale, the standardized values of venous VTI were multiplied by −1. Standardized variables were then summed to produce exploratory, combined index measures of retinal amyloid and retinal vascular features.

Differences in continuous variables between levels of CDR were assessed through one-way analysis of variance (ANOVA), with Bonferroni’s post-hoc test for the correction of multiple comparisons. Differences in the continuous variables between diagnostic scores were assessed using Student’s *t*-test. Linear regression was performed to assess the relationship between retinal vascular and retinal amyloid measures, as well as to assess the relationship between combined retinal vascular and amyloid counts, and cognitive parameters. All statistical analyses were performed using STATA v15.1 (StataCorp, College Station, TX, USA) with an a priori significance level of 0.05.

## 3. Results

Our study included a total of 34 subjects that presented to our Neurology clinic with cognitive concerns. Out of those 34, 29 had retinal images of sufficient quality to undergo both retinal amyloid and vascular analysis; their demographics and preexisting conditions are shown in Table 1. Mean MOCA was 26 (range of 4–32) and median MOCA was 27. Eleven subjects had a CDR of 0.5, 15 had a CDR of 1, and 3 had a CDR of 2. Regarding the formal neuropsychometric cognitive evaluation, 11 (37.93%) patients had normal cognition and 18 (62.06%) had impaired cognition (six with amnestic MCI, nine with multidomain MCI, two probable AD cases, and one with possible fronto-temporal lobar degeneration).

Linear regression analyses revealed that the venous branching angle correlated with the distal mid-periphery amyloid count (*p* = 0.03) and the arterial inflexion index correlated with the posterior pole amyloid count (*p* = 0.02). There were no associations between retinal vascular parameters and amyloid count in the proximal mid-periphery (Table S1).

**Figure 2:**
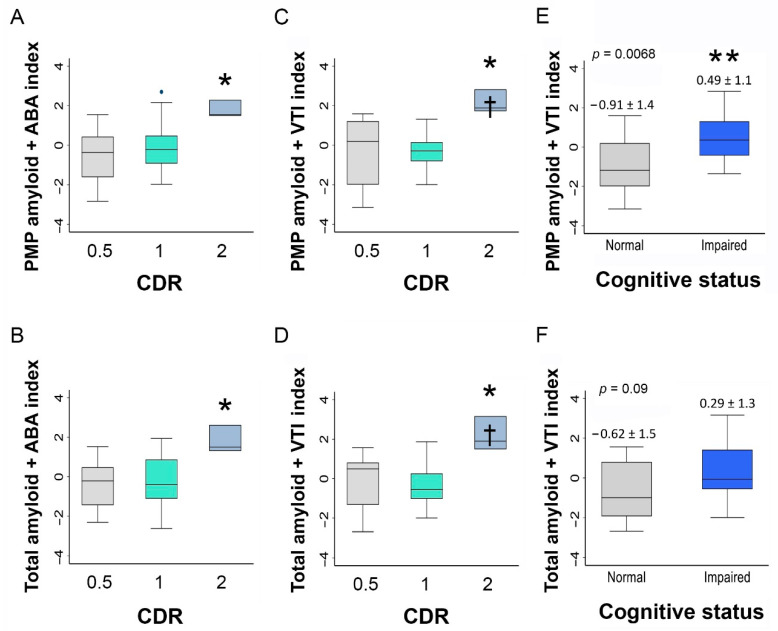
Combined retinal amyloid and vascular parameters in patients stratified by cognitive scores. Graphs illustrating the differences in the combined proximal mid-periphery amyloid-arterial branching angle index (**A**), total amyloid-arterial branching angle index (**B**), proximal mid-periphery amyloid-venous tortuosity index (**C**), and total amyloid-venous tortuosity index (**D**) when stratified by CDR score. Graphs illustrating the differences between the combined proximal mid periphery amyloid-venous tortuosity index (**E**) and total amyloid-venous tortuosity index (**F**) when stratified by the cognitive status. Bar graphs show the mean and deviation (* *p* < 0.05 and ** *p* < 0.01 by two-tailed paired Student’s *t*-test). Abbreviations: VTI, vessel tortuosity index; ABA, arterial branching angle; PMP, proximal mid-periphery; and CDR, Clinical Dementia Rating.

The analysis of retinal vascular and amyloid measures according to strata of cognitive function showed that the retinal PMP amyloid count and total amyloid count were significantly higher in the cognitively impaired compared to normal cognition participants (PMP: 144 ± 52 vs. 85 ± 32, *p* = 0.0012; total: 343 ± 90 vs. 247 ± 82, *p* = 0.04; Figure 1D,E and Table 2). There was no significant difference in the venous branching angle (*p* = 0.98) or arterial VTI (*p* = 0.53) across levels of CDR, whereas the arterial branching angle reached near significance (*p* = 0.066; Figure 1F). Venous VTI was significantly different across levels of CDR (mean ± SD of venous VTI values across increasing CDR categories: 0.13 ± 0.02, 0.13 ± 0.02, and 0.09 ± 0.02; *p* = 0.026; Figure 1G). Given these group differences and because of the independence of retinal vascular and retinal amyloid measures, the following combined amyloid-vascular indexes were calculated as exploratory variables: proximal mid-periphery amyloid count-venous VTI, total amyloid count-venous VTI, proximal mid-periphery amyloid count-arterial branching angle, and total amyloid count-arterial branching angle. One-way ANOVA revealed significant group differences in the VTI indices when compared according to the CDR level (Figure 2A–D). The combined proximal mid-periphery amyloid-venous VTI index was the only combined index measure exhibiting significant group differences when the cognitively impaired were compared to the cognitively normal subjects (0.49 ± 1.1 vs. −0.91 ± 1.4, *p* = 0.006; Figure 2E and Table 2).

We performed regression analyses to evaluate the correlations between retinal vascular geometric parameters and retinal amyloid counts with cognitive domain Z-scores. We found that the venous branching angle correlated with the WAIS-IV-digit span Z-score (Beta −0.045 (SE 0.015), *p* = 008). The total amyloid count correlated with the SF-36-MCS Z-score (Beta −0.004 (SE 0.002), *p* = 0.046), whereas the proximal mid-periphery amyloid count correlated with two verbal memory measures, namely CVLT-II Long Delay (Beta −0.009 (SE 0.003), *p* = 0.027) and WMS-IV LM-II (Beta −0.007 (SE 0.003), *p* = 0.028). The distal mid-periphery amyloid count correlated with non-verbal memory, RCFT Delayed Recall (Beta −0.01 (SE 0.005), *p* = 0.04), and SF-36-MCS (Beta −0.014 (SE 0.004), *p* = 0.004; Table 3).

**Figure 3:**
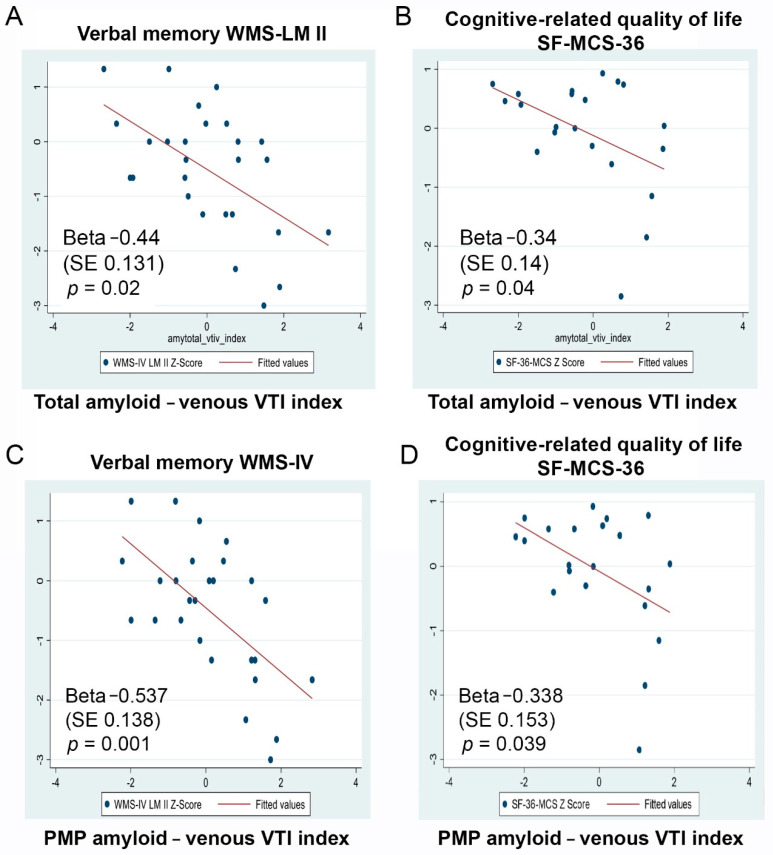
Retinal amyloid count combined with retinal venous VTI correlated with verbal memory and cognitive-related quality-of-life measures. Graphs illustrating the correlations between the combined total amyloid-venous tortuosity index and verbal memory (**A**) and cognitive-related quality-of-life Z-scores (**B**), and the correlations between the combined proximal mid-periphery amyloid-venous tortuosity index and verbal memory (**C**) and cognitive-related quality-of-life Z-scores (**D**). Abbreviations: WMS-IV LM II, Wechsler Memory Scale IV Logical Memory II; WMS-IV, Wechsler Memory Scale IV; SF-36 MCS, SF-36 Mental Component Score; PMP, proximal mid-periphery; and VTI, vessel tortuosity index.

The combined proximal mid-periphery amyloid-venous VTI index correlated with both verbal memory performance Z-scores (WMS-IV LM-II (Beta −0.537 (SE 0.138), *p* = 0.001) and CVLT-II Long Delay (Beta −0.370 (SE 0.176), *p* = 0.046)), as well as with the mental component of the cognitive-related quality-of-life score (SF-36-MCS (Beta −0.338 (SE 0.153), *p* = 0.039); Figure 3C,D). The combined total amyloid-venous VTI index correlated with WMS-IV LM-II (Beta −0.440 (SE 0.132), *p* = 0.003) and SF-36-MCS (Beta −0.302 (SE 0.141), *p* = 0.045; Figure 3A,B and Table 3).

## 4. Discussion

The main findings from this exploratory investigation of retinal fluorescence imaging are that retinal vascular features do not significantly correlate with retinal amyloid deposition in the proximal mid-periphery area; proximal mid-periphery retinal amyloid count correlates with verbal memory; and the combination of the retinal amyloid and venous tortuosity index into standardized index scores can provide a more comprehensive indicator of cognitive performance.

Microvascular damage is increasingly recognized as a critical initiator of vascular cognitive impairment and AD pathology [5,9,69]. Vascular dysregulation, leading to cerebral amyloid accumulation, and the link between cerebrovascular disease and dementia are explained by several mechanisms [4,5,70]. Pericyte loss and deficient vascular platelet-derived growth factor receptor-β signaling were identified in both the retinal and cerebral vasculature in subjects with MCI and AD [4,28]. Prior reports demonstrated that retinal vasculature may be used as a biomarker of early or preclinical dementia [71], and retinal microvascular abnormalities in MCI and dementia have been demonstrated using various retinal vasculature imaging modalities (e.g., retinal fundus photography [48,72,73], optical coherence tomography angiography [74,75], high-frequency flicker-light stimulation [51,76], and the retinal function imager [74]). Conversely, den Haan et al. [77] showed that retinal vascular measures did not differ between patients with AD and control participants, and venular tortuosity was smaller in subjects with greater white matter disease burden. Previous investigations have also shown a strong relationship between retinal vasculopathy and brain amyloid deposition [30]. Sharafi et al. [30] evaluated the relationship between retinal vascular statuses (vessel diameter and both tortuosity and spatial-spectral texture measures) using hyperspectral retinal imaging and CNS amyloid status (assessed with (18) F-florbetaben positron-emission tomography). They found that retinal venules of amyloid-positive subjects showed a higher mean tortuosity compared with the amyloid-negative subjects. This study suggested that the inclusion of metrics related to retinal vasculature and the surrounding tissue-related texture could improve the discrimination performance of the cerebral amyloid status [30].

As both retinal amyloid accumulation and retinal vascular pathology [28,34,35,36,37] are reported in patients with MCI, we explored the interplay between retinal vascular geometric measures and retinal amyloid burden using retinal fluorescence imaging. Prior studies showed that the retinal proximal mid-periphery area may be the target of amyloid quantification to reflect cerebral AD pathology, as it correlates with cognitive performance and hippocampal volume [35,37]. In this pilot cohort, we found that retinal vascular features correlated with amyloid deposition in the posterior pole and retinal distal mid-periphery area, but not with the proximal mid-periphery area. A possible explanation is that this investigation measured physical features of the retinal vasculature (e.g., branching angle and tortuosity index) and did not assess functional endpoints. Additionally, our quantitative vascular analysis could not target the smaller retinal blood vessels. The mechanisms driving vascular remodeling and amyloid deposition may occur at different rates, leading to the appearance of these clinical signs at different stages in disease progression. The investigation of subjects with mainly mild cognitive impairment in our cohort may explain why venous VTI was lower in subjects with worse cognition and why the other arterial or venous vascular parameters did not show any significant differences across cognitive strata. This hypothesis is supported by the lack of association between retinal vascular features and most of the neuropsychometric cognitive scores in our cohort. Conversely, retinal vascular measures did not correlate with any cognitive measures except for attention and concentration. The total and proximal mid-periphery retinal amyloid count correlated with verbal memory measures, while the distal mid-periphery amyloid count correlated with non-verbal memory measures. Interestingly, in this cohort with early cognitive disorders, subjects with higher amyloid counts and worse cognition levels had lower retinal venular tortuosity. The only combined index that discriminated between individuals with impaired cognition and normal cognition was the proximal mid-periphery amyloid count-venular VTI. This combined index was also associated with verbal memory and the ‘mental component’ summary of psychological functioning (SF-36 Mental Component Score). This latter finding reflects the association with cognitive-related psychological and emotional functioning. This appears to represent an exclusive contribution, as physical functioning status, as demonstrated by the SF-36 Physical Component Score, was not associated with amyloid or vascular retinal markers.

Two or more retinal vascular abnormalities were associated in a dose-response manner with an increased risk of disabling dementia in a prior study [49]. It is possible that combined amyloid–vascular indexes are better discriminators of cognitive function, with the potential for use as outcome measures in AD and mixed dementia trials. Our study is limited by a small sample size, heterogeneity, and the absence of genetic and CSF or brain amyloid biomarkers. Similarly, our patients were not evaluated for all ocular conditions other than a history of glaucoma. Due to the limited sample size, we could not adjust for the presence of traditional vascular risk factors or the presence of retinopathy, which are known contributors to retinal vascular geometric changes. Given the heterogeneity in the sample size across study groups, further confirmation of these preliminary results will be necessary in the future for specific groups of early AD and vascular and mixed dementias.

Our findings underscore the potential value of the exploratory amyloid-vascular indexes presented herein. Future investigations are warranted to explore the clinical utility of retinal fluorescence imaging in concert with combined amyloid-vascular index measures. More comprehensive cohort studies including a larger sample size and a greater range of disease severity among the participants could help to elucidate the stage at which retinal amyloid and/or retinal venular versus arterial impairments begin to develop in cognitive disorders. Given the cost and technical requirements of gold-standard methods for assessing cerebral amyloid deposition and vascular pathology, further validation of these retinal imaging methods could potentially yield greater accessibility to testing, thus facilitating more extensive clinical trials as well as improving the detection of early dementia.

## Figures and Tables

**Table 1:** Demographics and medical history of subjects in the combined retinal vascular and retinal amyloid analysis.

**N** (% female)	29 (55)
**Age** (years)	64 ± 6
	
**Preexisting health conditions,** N (%)	
Hypertension	11 (38)
Hyperlipidemia	15 (52)
Diabetes	3 (10)
Hyperthyroidism	8 (28)
Stroke/TIA	1 (3)
Heart disease/CAD/CHF	1 (3)
Smoking h/o	2 (7)

**Table 2:** Vascular and amyloid parameters stratified by the cognitive status.

Variable	Normal Cognition (*n* = 11)	Impaired Cognition (*n* = 18)	*p*
Age (years; mean ± SD)	66.92 ± 7.7	67.06 ± 7.41	0.954
Years of education (mean ± SD)	16.05 ± 1.43	15.59 ± 2.76	0.432
Arterial hypertension (%)	6 (54.4)	10 (55.55)	0.633
Dyslipidemia (%)	6 (54.4)	11 (61.11)	0.924
Diabetes Mellitus (%)	0 (0)	3 (16.66)	0.563
Hippocampal volume (cm^3^; mean ± SD)	7.82 ± 0.78	6.12 (±0.87)	0.051
Arterial branching angle (mean ± SD)	66.45 ± 13.0	63.3 ± 13.9	0.55
Venous branching angle (mean ± SD)	58.5 ± 6.1	59.0 ± 12.3	0.94
Arterial vessel tortuosity Index (mean ± SD)	0.15 ± 0.05	0.14 ± 0.06	0.42
Venous vessel tortuosity index (mean ± SD)	0.13 ± 0.02	0.12 ± 0.02	0.40
Arterial length (mean ± SD)	2687 ± 288	2706 ± 297	0.87
Venous length (mean ± SD)	2614 ± 270	2689 ± 276	0.50
Arterial vessel inflexion index (mean ± SD)	5.6 ± 0.80	5.5 ± 0.81	0.70
Venous vessel inflexion index (mean ± SD)	5.2 ± 0.91	5.3 ± 0.78	0.83
**Proximal mid-periphery amyloid count** **(mean ± SD)**	**85 ± 32**	**144 ± 52**	**0.0012**
Distal mid-periphery amyloid count (mean ± SD)	91.3 ± 63	93.1 ± 45	0.92
Posterior pole amyloid count (mean ± SD)	98 ± 60	106 ± 46	0.66
**Total amyloid count (mean ± SD)**	**247 ± 82**	**343 ± 90**	**0.04**
Combined proximal mid-periphery amyloid count–arterial branching angle index (mean ± SD)	−0.56 ± 1.3	0.27 ± 1.4	0.11
**Combined proximal mid-periphery amyloid count–venous tortuosity index (mean ± SD)**	**−0.91 ± 1.4**	**0.49 ± 1.1**	**0.0068**
Combined total amyloid count–arterial branching angle index (mean ± SD)	−0.27 ± 1.3	0.08 ± 1.4	0.51
Combined total amyloid count–venous tortuosity index (mean ± SD)	−0.62 ± 1.5	0.29 ± 1.3	0.09

**Table 3:** Retinal vascular and amyloid parameter predictors of cognitive domain measures.

Retinal Parameter	Cognitive Measures’ Z-Score	Beta (Std. Err)	*p*
Venous branching angle	WAIS-IV	−0.045 (0.015)	0.008
PMP amyloid-venous VTI	CVLT Long Delay	−0.37 (0.17)	0.04
Total amyloid-venous VTI	WMS LM-II	−0.44 (0.13)	0.03
PMP amyloid-arterial branching angle	WMS LM-II	−0.35 (0.16)	0.04
PMP amyloid-venous VTI	WMS LM-II	−0.53 (0.13)	0.001
Total amyloid-venous VTI	MCS	−0.30 (0.14)	0.04
PMP amyloid-venous VTI	MCS	−0.33 (0.15)	0.03
PMP amyloid count	CVLT Long Delay	−0.009 (0.003)	0.02
PMP amyloid count	WMS LM-II	−0.007 (0.03)	0.02
DMP amyloid count	RCFT 30 min Recall	−0.010 (0.005)	0.04
DMP amyloid count	MCS	−0.014 (0.004)	0.004
Total amyloid count	MCS	−0.004 (0.002)	0.04

Abbreviations: VTI, vessel tortuosity index; PMP, proximal mid-periphery; DMP, distal mid-periphery; WAIS, Wechsler Adult Intelligence Scale; CVLT, California Verbal Learning Test; WMS LM-II, Wechsler Memory Scale Logical Memory II; RCFT, Rey Complex Figure Test and Recall; MCS, Mental Component Score; and Std. Err, standard error.

## Data Availability

Data available upon request due to restrictions, e.g., privacy or ethical restrictions.

## Supplementary Materials

The following are available online at https://www.mdpi.com/article/10.3390/cells10112926/s1, Table S1: Retinal vascular parameter correlation with retinal amyloid measures.
