# Supplementary material for: Retinal Venular Tortuosity Jointly with Retinal Amyloid Burden Correlates with Verbal Memory Loss: A Pilot Study
Source: Cells. 2021 Oct 28;10(11):2926. doi: 10.3390/cells10112926 (PMC8616417; doi:10.3390/cells10112926)
Supplement: Supplementary file 1 [file cells-10-02926-s001.zip › cells-1299357-supplementary.pdf]

Table S1. Retinal Vascular Parameters correlation with of Retinal Amyloid.

Vascular Parameter	*Est (Std. Err)*	*p =*
	**Total Amyloid Count**	
Arterial BA	0.03 (1.2)	0.98
Venous BA	1.0 (1.3)	0.45
Venous VTI	-195 (305)	0.53
Arterial VTI	-320 (758)	0.68
Venous VII	9.49 (20.2)	0.64
Arterial VII	12.1 (20.3)	0.56
	**Proximal Mid-Periphery Amyloid Count**	
Arterial BA	0.07 (0.7)	0.92
Venous BA	0.12 (0.75)	0.88
Venous VTI	-95 (178)	0.60
Arterial VTI	-153 (442)	0.73
Venous VII	12.8 (11.6)	0.28
Arterial VII	3.40 (11.9)	0.78
	**Distal Mid-Periphery Amyloid Count**	
Arterial BA	-0.64 (0.69)	0.36
Venous BA	***1.6 (0.68)***	***0.03***
Venous VTI	75 (178)	0.68
Arterial VTI	241 (439)	0.59
Venous VII	-14.7 (11.4)	0.21
Arterial VII	-20.7 (11.2)	0.08
	**Posterior Pole Amyloid Count**	
Arterial BA	0.59 (0.74)	0.43
Venous BA	-0.71 (0.80)	0.39
Venous VTI	-175 (189)	0.36
Arterial VTI	-409 (468)	0.39
Venous VII	11.3 (12.5)	0.37
Arterial VII	***29.4 (11.4)***	***0.02***

Abbreviations: BA, branching angle; VTI, vessel tortuosity index; VII, vessel inflection index.
